# Intravenous push administration of anti-seizure medications

**DOI:** 10.3389/fneur.2024.1503025

**Published:** 2025-01-27

**Authors:** Raniah Aljadeed, Brian W. Gilbert, Tallib Karaze, Megan A. Rech

**Affiliations:** ^1^King Saud University, Riyadh, Saudi Arabia; ^2^King Saud University Medical City, Riyadh, Saudi Arabia; ^3^Department of Pharmacy, Wesley Medical Center, Wichita, KS, United States; ^4^Loyola University Medical Center, Maywood, IL, United States; ^5^Center of Innovation for Complex Chronic Healthcare, Edward Hines Jr. VA Hospital, Hines, IL, United States

**Keywords:** Antiepileptic drugs, antiseizure medications, status epilepticus, intravenous push, seizure

## Abstract

Intravenous push (IVP) administration of anti-seizure medications is becoming increasingly popular among emergency departments. IVP administration eliminates the need for compounding and preparation by the pharmacy department, as well as the need to gather infusion materials or set up a patient’s tubing and pump, all of which translate to faster drug administration. This is important given the time-sensitive nature of status epilepticus treatment. This review will discuss several anti-seizure medications, including phenytoin, fosphenytoin, valproic acid, levetiracetam, brivaracetam and lacosamide, for which evidence supports the safe and efficacious use of IV push administration.

## Introduction

Status epilepticus (SE) is a life-threatening medical emergency associated with mortality rates of up to 30% in adults and 3% in children (1). Multiple prognostic factors related to SE have been proposed, including the duration of the seizure and subsequent delays in treatment initiation (2, 3). Delay treatment initiation is associated with higher mortality and morbidity and worse functional outcomes (2).

Successful management of SE depends on rapid seizure cessation (4, 5). Management begins with benzodiazepines (BZD) as emergent initial therapy, particularly within the first 5 min of seizure onset (6). Given their efficacy and safety, these are Class 1 Level A recommendations for the management of SE. The most widely administered BZDs are diazepam, lorazepam, and midazolam. Directly following emergent therapy, intravenous (IV) urgent control therapy is administered within the first 5–10 min of seizure onset. *Antiseizure medications* (ASMs) used in urgent control therapy include fosphenytoin (FPT), phenytoin (PHT), valproic acid (VPA), levetiracetam (LEV), lacosamide (LAC), and brivaracetam (BRV). The primary goal of urgent control therapy is to halt seizures with optimal levels of ASM in patients who fail to respond to BZDs. It should be administered to all patients presenting with SE unless the cause of the SE is known and definitively correctable. Given the high mortality rate and time-dependent nature of SE, the rapid administration of ASM is critical for successful seizure termination.

Most non-BZD ASMs have historically been administered via intermittent intravenous piggyback (IVPB) infusions for 30–60 min (6). In addition to the infusion time, IVPBs are typically prepared in IV rooms within the pharmacy department, which adds logistical barriers, resulting in delayed administration. A meta-analysis of adult and pediatric patients with SE demonstrated median delays to treatment ranging from 69 min to 3 h (2). In contrast, intravenous push (IVP) administration has several advantages over IVPB. Although no precise definition of IVP exists, it generally refers to the direct manual administration of a medication using a syringe, usually under pressure, connected to an IV access device; this may include a manually administered IV bolus dose in an emergency (7). First, it obviates the need for reconstitution and preparation within the pharmacy department, in favor of bedside preparation. In addition, nurses no longer need to gather infusion materials and locate infusion pumps, prime IV lines, or program pumps. Consequently, the burden on pharmacy employees and nursing staff and material costs are reduced (Table 1). One study on cephalosporin administration found that 87% of the surveyed nurses preferred switching to IVP from IVPB administration (8). Furthermore, the faster administration of ASMs via the IVP route may translate in faster SE resolution. This review aimed to examine the safety and efficacy of administering select ASMs via IVP compared with the traditional IVPB route in the emergency department (ED).

**Table 1:** Advantages and disadvantages of the intravenous push administration method (1–5).

Advantages	Disadvantages
Faster drug administration	Injection site reactions (e.g., phlebitis, infiltration)
Eliminates need for pharmacist oversight of compounding and delivery to the patient care units	IVP administration is associated with a number of adverse effects, including local site reaction, dizziness, and somnolence
Reduces operational barriers by eliminating IV tubing and pump programming	
Lower cost of material and labor	Data regarding SE cessation, admission to the ICU, length of stay, and mortality have not been assessed
Conserves small volume fluids/parenteral solutions during critical shortages	
Medications can be stocked in automated dispensing cabinets in the ED	Increase time for nurses to be at the patient’s bedside during administration
Less fluid is used for IVP vs. IVPB, which could be important with fluid overloaded patients	

## Methods

A literature search was conducted on PubMed for randomized clinical trials, prospective and retrospective observational studies, case series, and case reports. The search keywords included: status epilepticus, epilepsy, seizures, emergency department, antiepileptic drugs, IVPB, IVP administration, PHT, FPT, LEV, LAC, VPA, and BRV. Table 2 summarizes the ASMs administered via the IVP, their dilution instructions, and their possible adverse effects.

**Table 2:** Intravenous push administration of ASM (1, 9–12, 14–16, 18, 26, 27, 31, 33, 34, 38, 39).

ASM	Typical loading dose	IVP administration			Adverse effects
		Max dose	Rate of administration	Dilution	
Phenytoin	20 mg/kg IV	≤ 250 mg	50 mg/min	Diluted to 25 mg/mL	Arrythmias, hypotension, cardiac arrest
Fosphenytoin	20 mg PE/kg IV	≤ 750 mg of PHT equivalents	150 mg/min	Diluted to 25 mg/mL	Arrythmias, hypotension, cardiac arrest
Valproic acid	20–40 mg/kg IV	40 mg/kg	3–6 mg/kg/min	Diluted with at least 50 mL	Transient pain at the injection, redness
Levetiracetam	1,000–3,000 mg IV or 60 mg/kg IV	4,500 mg	200–500 mg/ min	Undiluted, diluted 1:1 ratio	Dizziness, somnolence, fatigue, headache, agitation, local site reaction (redness, pain, burning and loss of IV access)
Brivaracetam	50–200 IV or ≥ 1.9 mg/kg	200 mg	2–15 min	Undiluted	Dizziness, headache, nausea
Lacosamide	200–400 mg IV	400 mg	80 mg/min	Undiluted	Hypotension, bradycardia

## Phenytoin and fosphenytoin

PHT inhibits fast voltage-gated sodium channels, thereby reducing neuronal firing and halting seizure activity (9). FPT is a prodrug of PHT that must be enzymatically hydrolyzed in a process that takes approximately 15 min. This time to conversion to an active drug is offset by the ability to administer more rapidly [150 mg PHT equivalents (mgPE)/min compared to 50 mg/min; Table 2]. The sodium channel blockade produced by these agents is a class IB antiarrhythmic; as such, cardiac arrhythmias are a primary safety concern. PHT is less water-soluble than FPT, necessitating the use of propylene glycol as a diluent, which carries the risk of cardiac arrhythmias and hypotension, especially if administered rapidly (10). Given the lower probability of infusion-related reactions and cardiac abnormalities with FPT, FPT is preferred over PHT in time-sensitive situations, such as SE. Despite these safety precautions, patients have been reported to experience bradyarrhythmia, hypotension, and cardiac arrest after treatment with both agents (11, 12). In the absence of time constraints, one may opt for PHT, given its cost-effectiveness as FPT increased costs by $40 per dose (13).

The much-anticipated Established Status Epilepticus Treatment Trial (ESETT) included 384 adults and children aged ≥2 years presenting to the ED with convulsive SE (14). Patients were randomized to receive one of three blinded treatments: LEV (60 mg/kg, maximum 4,500 mg), FPT (20 mg PE/kg, maximum 1,500 mg PE), or VPA (40 mg/kg, maximum 3,000 mg). These medications were secured within the unit in a medication box, which likely decreased the administration time compared to that in current practice, where many of these medications are compounded within the central pharmacies of institutions. Each study drug was administered using a programmed infusion pump over a 10-minute period. The drug concentrations were as follows: FPT, 16.66 mgPE/ml; VPA, 33.33 mg/mL; and, LEV, 50 mg/mL. Most patients had been seizing for 60 min prior to study enrollment in all three groups and had to be administered the study drug ≥5 min but ≤30 min from the last BZD dose within the ED before study drug infusion. This study demonstrated that FPT, LEV, and VPA had similar efficacies (45, 47% vs. 46%) and safety for the treatment of SE in the ED. One limitation worth highlighting is that the maximum dose of 1,500 mgPE may be suboptimal for patients weighing >75 kg, highlighting the issue of feasibility in administering larger doses. However, study drug administration was much quicker as IVPB in this trial compared to the to the administration time taking in traditional dosing recommendation.

Two randomized clinical trials, ConSEPT and EcLiPSE, exclusively involved children with convulsive SE in the ED who required second-line treatment. Participants were randomly selected to receive either LEV or PHT (15, 16). The ConSEPT trial enrolled 233 pediatric patients to receive PHT at 20 mg/kg via IV infusion over 20 min, diluted 1:4 with 0.9% sodium chloride and compared the outcomes with those receiving LEV (15). The EcLiPSE study was a multicenter, parallel-group, randomized, open-label superiority trial that included 286 pediatric patients presenting to the ED with convulsive SE who failed to respond to first-line treatment, where 152 and 134 patients were allocated to receive LEV and PHT, respectively (16, 17). PHT was administered at a dose of 20 mg/kg over 20 min, at a rate not exceeding 1 mg/kg/min (maximum dose, 2000 mg). The percentage of patients with seizure cessation was not statistically different between the two study groups (70% vs. 64%), with similar time from enrollment to seizure cessation in the LEV and PHT groups (35 min vs. 45 min; *p* = 0.2). Additionally, overall five serious adverse events were noted in the LEV and PHT groups but the difference between groups was not statistically significant (3 in the PHT group, 1 in the LEV group, and 1 in a patient who received both).

In addition, a prospective randomized study included 150 pediatric patients with SE, randomized into three equal groups: LEV, VPA, and PHT (18). No statistically significant differences were found between the groups in terms of seizure recurrence or control at 24 h. The PHT loading dose was 20 mg/kg diluted in 0.9% sodium chloride and infused at a rate less than 1 mg/kg/min.

IVP FPT has not been compared to IVPB FPT or PHT in time-sensitive situations in clinical studies. IVP FPT can be stocked in automated dispensing cabinets and administered as slow IVP for patients with SE. Phenytoin can cause tissue damage from extravasation, and the mechanism involves several factors, including its vehicle composition, strongly alkaline pH, and precipitate-forming abilities (19). Rapid infusion rates exceeding 25 mg/min, beyond the recommended infusion rate, are a significant risk factor for phenytoin extravasation and associated complications. To mitigate these risks, one approach is to administer FPT doses ≤750 mgPE, diluted to 25 mgPE/mL, over a 5-min period with the recommended administration rate and volume (30 mL). This process can be repeated in increments of 750 mgPE until the desired dose is reached. This obviates the need to wait for IV room preparation. Moreover, IVP is feasible for maintaining doses of both FPT and PHT. For instance, PHT can be administered at doses ≤250 mg diluted to a concentration of 25 mg/mL and administered no faster than 50 mg/min (Table 2). In addition, pushing doses ensures delivery of the entire dose during IVP administration in both maintenance and emergency situations, whereas with IVPB, some of the dose may remain in the tubing. One disadvantage of an all-IVP administration strategy (e.g., including loading and maintenance doses) is the potential for administration errors, leading to adverse effects (e.g., PHT administered faster than 50 mg/min). Therefore, education is a key component in switching to IVP PHT and FPT. However, further research is needed in this area.

In summary, for urgent SE treatment, we recommend IVP FPT at doses of up to 1,500 mg PE, at a rate not exceeding 150 mg PE/min. Maintenance doses of IVP PHT may save costs but should be implemented with clear infusion instructions to avoid adverse effects.

## Levetiracetam

LEV is indicated as an urgent control therapy in the management of SE, alongside FPT and VPA. Although the exact mechanism of action of LEV remains unknown, it has a high binding affinity for synaptic vesicle protein 2A (SV2A), thereby modulating the release of neurotransmitters, which plays an important role in the pathophysiology of epilepsy (20, 21).

Several studies with varying qualities of evidence and specific administration techniques have examined IVP administration of LEV at doses ranging from 10 to 60 mg/kg over 5 to 10 min (Table 2) (14–16, 22–27). LEV appears to be well tolerated as an undiluted IVP at doses ranging from 250 to 4,500 mg at a rate of 200–500 mg/min (26–28). A separate study investigated the safety of undiluted IVP administration of LEV at doses up to 2000 mg in patients without SE (29). Overall, 60 doses were administered via IVP at a rate of ≥200 mg/min, and 100 doses were administered via IVPB. The study revealed a significant difference in the duration of time to LEV administration between the IVP and IVPB groups (28 vs. 80 min; *p* < 0.00001). In terms of safety, no concentration-related adverse effects were observed in any group. Moreover, no significant differences in behavior-related adverse effects were observed between the groups.

Other studies examining faster infusion rates of LEV revealed that rates up to 500 mg/min were effective and tolerated and had minimal side effects (22, 24). LEV doses were diluted at a ratio of 1:1 or mixed with 100 mL of 0.9% sodium chloride or 5% dextrose. The first study was a randomized, placebo-controlled trial by Ramael et al. that included 48 healthy adults aged 18–55 years (22). The patients received either a placebo or one of six different LEV doses via IV infusion. Of the six LEV groups, the highest administration rates fell into one of three groups: 300, 400, and 500 mg/min. The second study by Wheless et al. was an open-label, prospective, single-center study involving 45 patients aged 4–32 years (24). All patients were diagnosed with either partial-onset seizures or generalized epilepsy. LEV was administered at 200 mg/min, 400 mg/min, and 500 mg/min. A pharmacokinetic study of 48 healthy adults found a comparable half-life, total body clearance, and volume of LEV distribution across varying infusion rates (15 min for doses >2,500 mg – 4,000 mg and 5 min for doses ≤2,500 mg) (22).

In addition, the previously described EcLiPSE study administered LEV at 40 mg/kg up to 2,500 mg via IV route over 5 min, diluted with 0.9% sodium chloride to a maximum of 50 mg/mL (16). Additionally, the previously described ConSEPT trial administered LEV at 40 mg/kg up to 3,000 mg, diluted 1:1 with 0.9% sodium chloride, intravenously over 5 min (15). In the ESETT trial, LEV was administered as a diluted infusion over 10 min at a dose of 60 mg/kg up to 4,500 mg (14).

Overall, undiluted IVP administration of LEV appears to be safe over a wide dosage range, from a high dosage for the rapid treatment of seizures to a lower maintenance dosage. Premixed bags are an alternative option, although they are limited to certain doses. Utilizing premixed bags may lead to the delayed administration of higher doses (e.g., loading doses). Given its ability to be administered quickly with minimal adverse effects, IVP LEV is preferable over IVPB for the treatment of SE. They can easily be stored in automated dispensing cabinets for rapid bedside access.

## Lacosamide

LAC selectively inactivates slow-voltage-gated sodium ion channels, which in turn reduces the nerve conduction responsible for producing seizures (30). Given its relatively acidic pH of 3.5–5, initial studies diluted LAC with 50–100 mL of 0.9% sodium chloride, 5% dextrose, or lactated Ringers solution. However, recent literature supports the administration of undiluted IVP LAC at 80 mg/min (31–33) (Table 2).

A retrospective cohort study compared the administrations of undiluted IVP-LAC (n = 78) and IVPB-LAC (n = 88) for urgent seizure control (32). The time to administration was significantly faster in the IVP group (35 min vs. 109 min; *p* < 0.001). Adverse effects were rare overall and similar between groups; they included hypotension (8% vs. 10.3%; *p* = 0.61), and bradycardia (2.6% vs. 2.3%; *p* > 0.99). No infusion-related reactions were reported. However, outcomes such as the time to resolution of SE, admission to the intensive care unit, and mortality were not collected and should be evaluated in future studies. Regarding safety endpoints, in a retrospective cohort study comparing 102 patients receiving undiluted IVP LAC with 73 patients receiving IVPB infusion, adverse effects were rare overall and similar between groups: bradycardia (0.2% IVP vs. 1.1% IVPB; *p* = 0.07) and hypotension (3.2% IVP vs. 1.6% IVPB; *p* = 0.12) (31). Infusion-related reactions were uncommon following IVP administration, occurring in <2% of administered doses.

LAC is a safe and effective treatment option in both emergency and non-emergency settings. Doses of up to 400 mg of LAC can be administered over 5 min, making this agent ideal for storage in automated dispensing cabinets in the ED. This also eliminates the need for pharmacists to sign for the controlled substance, resulting in a shorter time to the first dose availability in patient care areas (31). The Neurocritical Care Society status epilepticus guidelines list lacosamide as an option for management in refractory patients; however, there has been increased utilization in various cohorts of patients with seizures in recent years, with a favorable side effect profile and data to support its non-inferiority in non-convulsive status epilepticus in LAC doses administered over 30 min as an infusion (34, 35). Given the temporal relationship between the approval of LAC, guideline iterations, and literature supporting rapid undiluted administration, it is reasonable to expect LAC to have a more prominent role in subsequent guideline updates.

## Valproic acid

VPA has several mechanisms of action, with no single mechanism accounting for all its effects. It inhibits gamma-aminobutyric acid degradation and stimulates its synthesis, which is involved in the management of seizure generation and propagation (36, 37). Second, it inhibits the action of *N*-methyl-D-aspartate and glutamate, a positive neurotransmitter, resulting in the suppression of seizure activity. Furthermore, VPA reduces the high firing frequency of neurons by blocking the voltage-gated sodium, potassium, and calcium channels, resulting in reduced seizure activity (37). Although VPA exerts a myriad of effects, the exact mechanism contributing to the cessation of seizures is yet to be elucidated (36).

Despite the availability of VPA for over 40 years, surprisingly, limited evidence supports its use as a rapid IVP-administered medication. The commonly reported effective doses for bolus and infusion are 15–45 mg/kg (6 mg/kg/min), which translates to infusion times ranging from 2.5 to 7.5 min (38). In terms of safety, multiple studies showed a low incidence of adverse events overall (<10%), including dizziness, thrombocytopenia, and mild hypotension. All effects appeared to be independent of the infusion rate. Additional studies, although they were not conducted in patients with SE, demonstrated that the rapid administration of undiluted valproate was well tolerated without any significant neurological, hepatic, cardiovascular, or local adverse effects, supporting its use in emergency situations (39). The aforementioned ESETT trial administered valproate at 40 mg/kg by a programmed infusion pump over a 10-min period with a maximum dose of 3,000 mg (14).

In summary, although the ESETT study did not use IVP administration, it may be feasible to administer VPA at 6 mg/kg/min. Further research is needed to investigate the efficacy and safety of higher administration rates. In a systematic review and meta-analysis, VPA was found to be the most successful in terminating BZD-resistant SE as compared to three other agents (LEV, PHT, and phenobarbital) (40). Given its broad applications for the cessation of seizures and the high rate of termination of SE, further research is warranted on its administration via IVP in treating patients with SE.

## Brivaracetam

BRV is an LEV analog used to treat SE. Although its precise mechanism of action is unknown, it has a greater affinity for the glycoprotein SV2A than LEV (41, 42). Moreover, it is more lipophilic, allowing it to cross the blood–brain barrier more efficiently and reach its target concentration 3–6 times faster than LEV. Whether these pharmacokinetic differences translate to enhanced efficacy of SE is currently unknown (43).

Although IV BRV was not labeled for SE treatment, the loading dose ranges from to 25–400 mg (43, 44). There is limited guidance regarding the use of BRV specifically as an IVP medication for SE in the ED, and the administration rates range from to 2–15 min (41). A retrospective case series of BRV for SE included seven patients receiving a median of four ASMs prior to BRV administration and found that outcomes according to the Glasgow Outcome Scale were improved in 86% of patients (45). A subsequent retrospective multicenter registry of patients with SE who were treated with BRV included 43 patients. A dose of 1.82 mg/kg was associated with faster response times in SE control (44). This result is consistent with that of another retrospective study that showed that doses of at least 1.9 mg/kg yielded a greater probability of SE cessation without the need for further treatment (43). Further research is needed to elucidate the optimal dose of BRV for SE, but doses ≥1.9 mg/kg via IVP appear to be most effective. In addition to the exact dosing recommendations, studies on the early use of BRV for SE and determining its place in therapy compared with other ASMs are lacking. Currently, BRV is not included in the SE treatment guidelines, but its optimal pharmacokinetic properties make it a promising treatment option. Similar to LAC, this agent can be stored in automated dispensing cabinets in the ED. Because BRV is a controlled substance, stocking it may result in less time to administration in patient care areas.

## Practical implications

The Institute for Safe Medication Practices (ISMP) developed a set of guidelines on safe practices for preparing and administering adult IVP medication (7). These guidelines outline the proper preparation of IVP medications such that a ready-to-administer form can be provided to patients. If rapid administration is required, dilution can be performed immediately prior to administration in a clean, uncluttered, and separate location with clear instructions indicating the type and volume of the diluent. Any prepared syringes should be clearly labeled; maintaining a steady supply of blank, ready-to-apply labels in the patient area helps facilitate adherence to these practices.

In addition, safe IVP practices involve adhering to clear instructions regarding specific rates of administration on syringe labels as part of the original order; these instructions are provided in the medication administration record (7). Instead of IVP rates of “slow” or “fast” injection which can be rather ambiguous, clear, precise rates of administration should be noted. These specific rates should also be included as part of the medication order in the order sets. Additionally, to facilitate the proper preparation of IVP medications, nurses should be educated on the dilution and rates of administration of IVP medications. Periodic competency evaluations should be performed.

To assist in this step, emergency medicine pharmacists can provide valuable assistance at the patient’s bedside. They facilitate the safe delivery of ASM via IVP, assist in mixing medications at the bedside, optimizing treatment decisions, and setting up medications on pumps to ensure appropriate delivery and administration. The American College of Emergency Physicians (ACEP) and the American College of Medical Toxicology (ACMT) released statements emphasizing the value of emergency medicine pharmacists and the significant positive impact of their presence on emergency medicine teams (46, 47). Published data support the inclusion of an emergency medicine pharmacist as part of the medical team for serving critical care patients; the presence of an ED pharmacist is associated with a reduction in the time to antibiotic administration for patients with sepsis, a reduction in the time to thrombolysis in patients with acute ischemic stroke, and a reduction in the time to administration of ASMs (48–51). Lastly, this study focuses on a specific approach to IVP ASMs administration that may align with some local practices. We recognize that methods such as bedside preparation of piggyback fluids, use of pre-mixed bags, or centralized pharmacy preparation are common in other countries. These variations stem from differences in healthcare systems, regulatory frameworks, and resource availability.

## Conclusion

IVP administration of ASMs is gaining popularity in EDs because of its ease of preparation, ability to be stored in automated cabinets proximal to patients with SE, and elimination of pumps and supplies, thereby reducing the time to administration. An important consideration remains for doses that would require more than 5 min for IVP administration. These cases will require bedside time from nurses, which can be a critical factor in hospitals with a shortage of personnel. Several studies have assessed the safety and efficacy of IVP administration of FPT, VPA, LEV, LAC, and BRV. Although the IVP route of administration results in a faster time to administration of ASMs than the IVPB route, additional studies are needed to determine if this route of administration improves outcomes such as mortality and length of stay in patients with SE.
